# Deciphering Host Genomic Pathway Landscapes in Severe COVID‐19 by Exome Sequencing of a Large Admixed Brazilian Cohort

**DOI:** 10.1002/jmv.71193

**Published:** 2026-11-02

**Authors:** Aléxia Stefani Siqueira Zetum, Danielle Ribeiro Campos da Silva, Vinícius do Prado Ventorim, Felipe Ataides Mion, Felipe dos Santos Passarela, Patrícia Brasil, Túlio de Lima Campos, Bartolomeu Acioli‐Santos, Flávio Rosendo da Silva Oliveira, Raquel Silva dos Reis Trabach, Lorena Souza Castro Altoé, Débora Dummer Meira, Iúri Drumond Louro

**Affiliations:** ^1^ Science Biology Department Federal University of Espírito Santo Vitória Espírito Santo Brazil; ^2^ Haemostasis Laboratory Hemotherapy and Hematology Center of Espírito Santo Vitória Espírito Santo Brazil; ^3^ Oswaldo Cruz Foundation Rio de Janeiro Rio de Janeiro Brazil; ^4^ Bioinformatics Center, Aggeu Magalhães Institute, Oswaldo Cruz Foundation Recife Pernambuco Brazil; ^5^ Department of Virology Aggeu Magalhães Institute, Oswaldo Cruz Foundation Recife Pernambuco Brazil; ^6^ Federal Institute of Education, Science and Technology of Pernambuco Recife Pernambuco Brazil

**Keywords:** complement activation, epithelial integrity, host genomics, immunometabolism, mitochondrial dysfunction

## Abstract

The host genetic factors that resulted in the heterogeneous clinical outcomes of COVID‐19 remained poorly understood, especially in admixed populations that are underrepresented in global genomic studies. In this study, we investigated the genetic landscape associated with COVID‐19 severity using whole‐exome sequencing data from 325 Brazilian individuals recruited between 2020 and 2023. To prioritize variants related to disease severity, we applied a machine learning framework combining Support Vector Machine – Recursive Feature Elimination and SHAP interpretation. This approach encompasses 25 variants that contributed to the model's discrimination between severe and non‐severe cases (AUC = 0.94) in our cohort. Subsequently, logistic regression analyses adjusted for age, sex, principal components of ancestry, and significant comorbidities confirmed several loci associated with severity after correction for multiple testing (FDR < 0.05). The prioritized genes were grouped into biological axes, including mitochondrial bioenergetics, neuroimmune signaling, complement activation, and epithelial structural integrity. Variants in genes such as *MUC16, SLC4A2, LAPS1, ARRDC2*, and *CCSER1* showed associations with disease severity. Furthermore, functional annotation using CADD and regulatory exploration using GTEx suggested that several loci may influence gene regulation in lung, immune, and neural tissues. Instead of implicating single causal variants, our findings highlight pathway‐level genetic architectures that may contribute to the variability of the host response to COVID‐19. These findings provide exploratory insights into potential host genetic pathways that may be involved in disease variability in admixed environments. However, replication in independent cohorts and functional validation are still needed.

**Abbreviations**
ACE2: angiotensin‐converting enzyme 2
AE2: anion exchanger 2 (encoded by SLC4A2)
ARRDC2: arrestin domain‐containing protein 2
ATP: adenosine triphosphate
C3: complement component 3
CAAE: certificate of Presentation of Ethical Appreciation
CatL: cathepsin L
CCSER1: coiled‐coil serine‐rich protein 1
CNS: central nervous system
COL19A1: collagen type XIX alpha 1 chain
COVID‐19: coronavirus disease 2019
ER: endoplasmic reticulum
ERK: extracellular signal‐regulated kinase
ERRα: estrogen‐related receptor alpha
ETC: electron transport chain
GABA: gamma‐aminobutyric acid
GAD2: glutamate decarboxylase 2
IFN‐I: type I interferon
ITPR1: inositol 1,4,5‐trisphosphate receptor type 1
K29: lysine 29
LASP1: LIM and SH3 protein 1
LINC01118: long intergenic non‐protein coding RNA 1118
MEK: mitogen‐activated protein kinase kinase
mtROS: mitochondrial reactive oxygen species
MUC16: mucin 16
NaV1.7: voltage‐gated sodium channel Nav1.7 (encoded by SCN9A)
NPC1: Niemann‐Pick C1 protein
NRF1: nuclear respiratory factor 1
NRF2: nuclear factor erythroid 2‐related factor 2
NRP2: neuropilin‐2
ODAD1: outer dynein arm docking complex subunit 1
pHi: intracellular pH
PLA2G4D: phospholipase A2 group IVD
PODN: Podocan
PPARGC1A: peroxisome proliferator‐activated receptor gamma coactivator 1‐alpha
PTPN5: protein tyrosine phosphatase non‐receptor type 5 (STEP)
Raf: rapidly accelerated fibrosarcoma kinase
RAI14: retinoic acid induced 14
RNF220: ring finger protein 220
RNF222: ring finger protein 222
ROS: reactive oxygen species
SARS‐CoV‐2: severe acute respiratory syndrome coronavirus 2
SCN9A: sodium voltage‐gated channel alpha subunit 9
SESA: Health Department of the Government of Espírito Santo
SLC23A2: solute carrier family 23 member 2 (SVCT2)
SLC4A2: solute carrier family 4 member 2 (Anion Exchanger 2)
SNED1: sushi, nidogen and EGF‐like domains 1
STAT1: signal transducer and activator of transcription 1
STEP: striatal‐enriched protein tyrosine phosphatase (PTPN5)
SVCT2: sodium‐dependent vitamin C transporter 2 (encoded by SLC23A2)
TLR: toll‐like receptor
TMEM11: transmembrane protein 11
TMPRSS2: transmembrane serine protease 2
TRIM6: tripartite motif‐containing protein 6
VitC: vitamin C (ascorbic acid)

## 1 Introduction

Advances in population genomics have led to the identification of host genetic variations that may be associated with different outcomes observed in severe acute respiratory syndrome coronavirus 2 (SARS‐CoV‐2) infection. Genes such as *ACE2, TMPRSS2, SLC6A20*, and elements of the HLA system in the reaction to the virus have drawn attention from whole‐exome sequencing and Genome‐Wide Association Studies (GWAS) [1]. Multicenter analyses that found loci linked to respiratory failure, intensive care unit admission, and multisystem inflammatory syndrome have provided additional evidence for these conclusions. Additionally, variations of innate immunity‐related genes, such as *TLR7, TLR3, IRAK1*, and *OAS1‐3*, have been linked to a higher risk of unfavorable outcomes [2].

In Brazilian cohorts, various genetic analyses have identified specific variants and HLA alleles with potential influence on the antiviral immune response [3]. While there has been significant growth in the coronavirus disease 2019 (COVID‐19) genetic literature, research remains unevenly distributed, with heavy reliance on populations of European ancestry, and the majority of SARS‐related studies also involving East Asian participants [4].

In this study, we applied a Support Vector Machine (SVM) framework to whole‐exome data from Brazilian individuals with confirmed COVID‐19. The model prioritized 25 variants and encompassed pathways related to innate immunity, mitochondrial metabolism, and neuroinflammatory signaling. The patterns identified here appear to reflect biological axes previously associated with COVID‐19, although they need to be interpreted from an exploratory perspective.

In the following section, we present the results of our study, focusing on the genes and pathways prioritized in the cohort. Our results outline functional structures involving different pathways, particularly those of innate immunity, mitochondrial bioenergetics, and neuroinflammatory signaling. The study design, cohort selection, data processing, and analysis workflow are described in detail in the following sections.

## 2 Materials and Methods

### 2.1 Patients and Control Group

This research was approved by the Human Research Ethics Committee of the Health Sciences Center/UFES, under number CAAE: 37094020.6.0000.5060. A total of 325 participants were included in the cohort. Participants were recruited between November 2020 and December 2023 through collaborating healthcare institutions in the Greater Vitória metropolitan area, Espírito Santo, Brazil, including Dr. Jayme Santos Neves State Hospital [5]. All volunteers signed a written informed consent form and only individuals who were not vaccinated against SARS‐CoV‐2 prior to infection were included. SARS‐CoV‐2 infection was confirmed by either real‐time reverse transcription polymerase chain reaction (RT‐PCR) or rapid antigen testing. Participants were categorized as severe if they met the criteria for severe or critical disease, such as oxygen saturation below 90%, respiratory rate above 30 breaths per minute, or need for additional oxygen, mechanical ventilation, or admission to an intensive care unit. Non‐severe patients were those who had mild to moderate illness and showed no symptoms of hypoxia or severe respiratory distress [6]. Whole blood samples were kept refrigerated (2°C–8°C) until DNA extraction.

### 2.2 Genomic DNA Extraction

The samples were processed using the QIAamp Mini Kit (QIAGEN®, Hilden, Germany), and the concentration and purity of the DNA were measured using the NanoDrop® (Thermo Scientific, USA) and Qubit® (Life Technologies, USA). The final concentration used for sequencing was 6.67 ng/µL.

### 2.3 DNA Library Preparation and Genotyping

The National Institute of Cardiology (INC) carried out whole‐exome sequencing using the Illumina DNA Prep with Enrichment protocol. The Qubit dsDNA BR Assay Kit was used for quantification, and the Agilent Bioanalyzer was used to measure fragment size and distribution.

### 2.4 Data Preprocessing

We utilized Illumina's DRAGEN software (San Diego, California, USA). The reference genome hg38‐altmaskedv3‐graph‐enabled and the Illumina Exome Panel v1.2 (CEX) was used. Identified variants were annotated using Nirvana software. Following sequencing, Illumina's BaseSpace Sequence Hub platform was used to store the FASTq, Binary Alignment/Map (BAM), Index File (BAI), and Variant Call Format (VCF) files.

### 2.5 Quality and Variant Filtering

The quality of the VCF files generated was evaluated using DISCVRSeq (v1.3.62) [7]. Variant filtering was performed with VCFtools (v0.1.16) [8], including minor allele frequency (MAF ≥ 0.05), minimum variant quality score (Phred ≥ 20), minimum read depth (DP ≥ 5), and a maximum of 10% missing genotypes per variant. Hardy–Weinberg equilibrium testing was applied exclusively to the control group (*p* > 1 × 10^−5^ threshold), and variants deviating from equilibrium were excluded. Variant‐level quality metrics were further assessed using VariantQC. Only variants marked as PASS were retained for downstream association analyses.

### 2.6 Admixture

Admixture analysis and estimation of the number of populations were conducted using ADMIXTURE (v1.3.0) (K = 2–5 clusters) [9]. The 1000 Genomes Project data was used as a reference for ancestry analysis and was incorporated into the FRAPOSA algorithm [10] with default settings. The Gencode.exome database and the EthSEQ tool [11] were used for principal component analysis (PCA) visualization and ancestry classification. The obtained PCAs were subsequently included as covariates in the logistic regression (LR) models in order to account for population stratification.

### 2.7 Analyses with Machine Learning Algorithms

The SVM‐RFE framework was primarily used as a dimensionality reduction tool, with the goal of prioritizing variants within our cohort. Missing genotypes were imputed using the mode after columns with more than 10% missing data were eliminated [12]. To evaluate internal model performance, the AUC metrics of the ROC curve, complemented by accuracy and the F1 score, were obtained. We verified the contributions of single nucleotide polymorphisms (SNPs) to the model's predictions using SHapley Additive exPlanations (SHAP).

### 2.8 Statistical Analysis

The chi‐square test was used to analyze categorical variables (Python 3.12.11). LR analyses were performed using PLINK v1.9 under an additive genetic model. Odds ratios (OR) and 95% confidence intervals (CI) were estimated. Analyses were adjusted for sex, age, principal components of ancestry (PCA) and significant comorbidities. *p*‐values were corrected for multiple tests using the Benjamini–Hochberg false discovery rate (FDR‐BH) procedure. Variants with FDR‐adjusted *p*‐values < 0.05 were considered statistically significant.

### 2.9 Functional Annotation of Prioritized Variants

To provide biological context to the prioritized variants we analyzed the predicted deleteriousness using the Combined Annotation‐Depletion Dependent Framework (CADD, v1.6). PHRED scale scores were obtained for all variants selected by the model to verify possible functional constraints, including potential non‐coding regulatory effects. In parallel, we also explored publicly available quantitative expression trait loci (eQTL) and Splicing QTLs (sQTL) data from the GTEx database (v8).

## 3 Results

### 3.1 Sociodemographis and Clinical Characteristics

In the descriptive analysis of the cohort (*N* = 325), 48.9% of participants had a severe clinical condition. Sex distribution differed significantly between severity groups (FDR‐adjusted *p* = 0.001), with a higher proportion of males observed among severe cases. Age group was also significantly associated with disease severity (FDR‐adjusted *p* < 0.001). Educational level showed a significant association with severity (FDR‐adjusted *p* = 0.014). Regarding comorbidities, chronic lung disease (FDR‐adjusted *p* = 0.006), chronic cardiovascular disease (FDR‐adjusted *p* < 0.001), diabetes mellitus (FDR‐adjusted *p* < 0.001), and obesity (FDR‐adjusted *p* < 0.001) were significantly associated with severe COVID‐19 within this cohort (Table 1).

**Table 1:** Clinical and sociodemographic profile of the study population by disease severity.

		*N* = 325	%	*p‐value	FDR
Clinical spectrum	Non‐Severe	166	51.08	—	—
	Severe	159	48.92		
Sex	Female	183	56.31	0.001*	0.001*
	Male	142	43.69		
Age group	Under 39 years	110	33.85	< 0.001*	< 0.001*
	40‐49 years	87	26.77		
	Above 50 years	128	39.38		
Schooling	No higher schooling	180	55.38	0.014*	0.014*
	Higher schooling	126	38.77		
	S/I	19	5.85		
Ethnic group	Non‐White	136	41.85	0.579	—
	White	189	58.15		
Physical activities	No	145	44.61	0.080	—
	Yes	179	55.08		
	S/I	1	0.31		
Smoking	No	237	72.92	0.521	—
	Yes	33	10.15		
	S/I	55	16.92		
Chronic lung disease	No	281	86.46	0.006*	0.006*
	Yes	44	13.54		
Chronic cardiovascular disease	No	225	69.23	< 0.001*	< 0.001*
	Yes	100	30.77		
Diabetes mellitus	No	286	88	< 0.001*	< 0.001*
	Yes	39	12		
Obesity IMC	No	191	58.77	< 0.001*	< 0.001*
	Yes	129	39.69		
	S/I	5	1.54		

*Note:* Values are presented as absolute frequency (*n*) and percentage (%). Associations between categorical variables and COVID‐19 severity (non‐severe vs. severe) were evaluated using the chi‐square test. Values in bold and marked with an * indicate statistical significance (*p*‐value < 0.05).

Abbreviations: BMI, body mass index; S/I, missing or not informed data.

### 3.2 Variant Filtering and Genomic Landscape

Initially, a total of 651,652 loci was identified through raw sequencing data analysis (DRAGEN). Most sequenced variants were classified as PASS (70.3%). Among the filtered variants, 26.5% were annotated as MosaicLowAF, while a smaller proportion was flagged due to depth‐ or quality‐related filters, including LowDP (0.24%). Variants that did not meet DRAGEN's stringent quality thresholds represented less than 0.1% of the calls. After filtering, 192,876 variants remained, predominantly SNPs (97.9%), followed by deletions (0.9%) and insertions (0.6%). Other additional filters described in this methodology reduced the data set to approximately 8000 SNPs for subsequent analysis.

### 3.3 Variant Prioritization and Internal Separation Pattern

In our study, 25 variants contributed to model discrimination between severity groups (accuracy: 87%; F1‐score: 86%; AUC: 94%).

Among the 25 prioritized variants, 13 showed positive SHAP contributions: *LINC01118* ‐ rs2289933, *TRIM6* ‐ rs3751006, *ARRDC2* ‐ rs7259041, *SNED1* ‐ rs7571117, *CCSER1* ‐ rs6843110, *PODN* ‐ rs1769316, *RNF222* ‐ rs12601265, *COL19A1* ‐ rs7762409, *GAD2* ‐ rs2839671, *PLA2G4D* ‐ rs4924618, *RAI14* ‐ rs1048944, *COLEC11* ‐ rs3811527, *MUC16* ‐ rs12462963.

While 12 showed negative SHAP contributions: *SLC23A2* ‐ rs1110277, *SLC4A2* ‐ rs2303937, *ITPR1* ‐ rs2306875, *TMEM11* ‐ rs3744223, *C3* ‐ rs2287845, *LASP1* ‐ rs525989, *PTPN5* ‐ rs1550870, *NPC1* ‐ rs1367083, *SCN9A* ‐ rs6747673, *ODAD1* ‐ rs8107712, *COLEC11* ‐ rs3811527, *NRP2* ‐ rs849526 (Figure 1).

**Figure 1:**
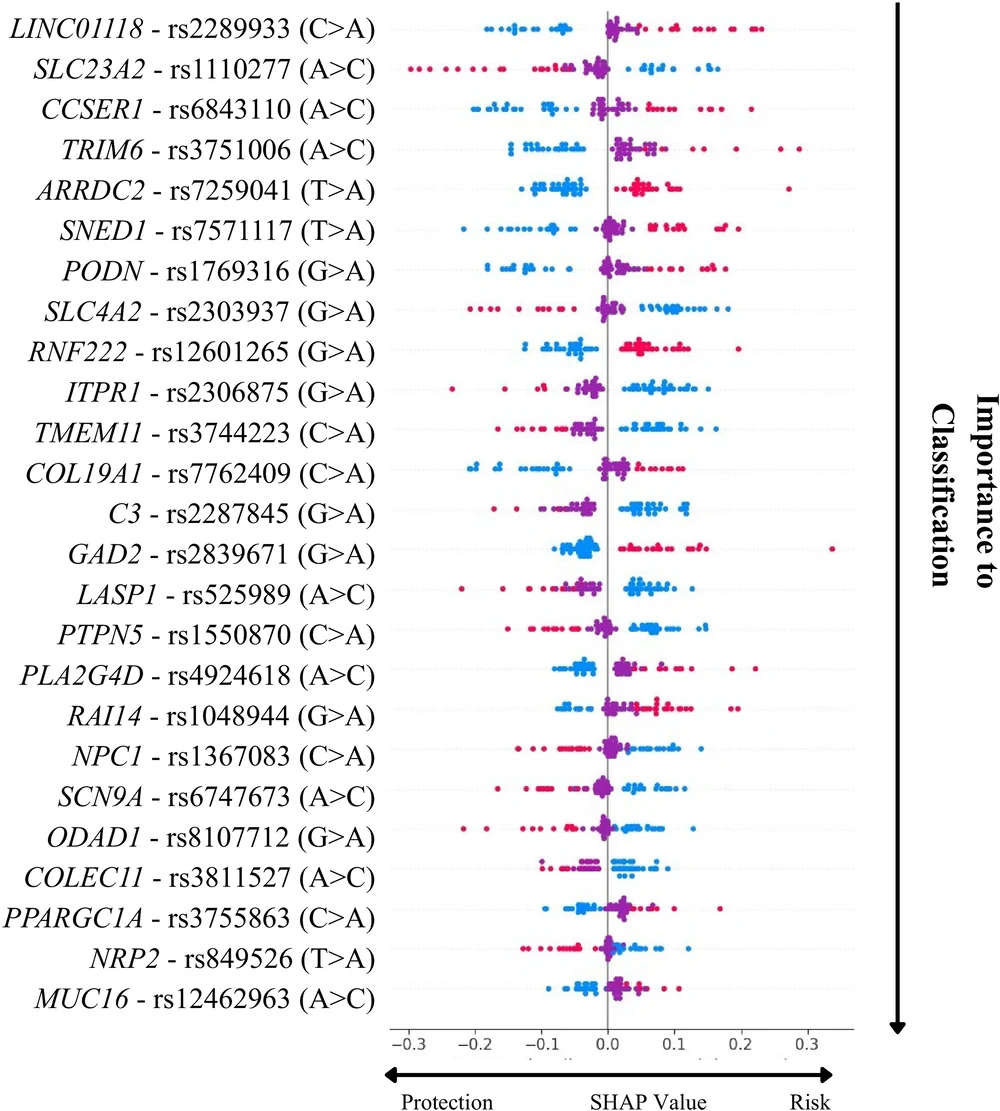
Variant prioritization and contribution patterns identified by SVM–RFE. Figure 1 demonstrates the directional contribution of the 25 prioritized variants to the model predictions (SHAP). Each point represents an individual, and each color represents a genotype: genotypic: blue indicates the reference genotype (wild type), purple indicates the heterozygous state, and magenta indicates the homozygous alternative genotype (variant). We can also observe the horizontal shift from zero, which reflects the magnitude and direction of each variant's contribution to the classification. In general, positive values indicate a contribution to the severe classification and negative values to the non‐severe classification. All variants are ranked vertically by overall importance to the classification, from highest (above) to lowest (below).

### 3.4 Independent Logistic Regression Validation of Classifier‐Selected Variants

After FDR‐BH correction, multiple loci remained significantly associated with COVID‐19 severity (FDR ≤ 0.05) (Table 2). Variants associated with increased odds of severe disease included *MUC16* ‐ rs12462963, *ARRDC2* ‐ rs7259041 and *CCSER1* ‐ rs6843110. Conversely, variants associated with decreased odds of severe disease included *SLC4A2* ‐ rs2303937, *LASP1* ‐ rs525989 and *PTPN5* ‐ rs1550870.

**Table 2:** Logistic regression results for classifier‐selected variants (additive model).

*SNP*	*OR*	*L95*	*U95*	*p‐value	FDR_BH*
*MUC16* ‐ rs12462963 (A > C)	2.146	1.371	3.359	**< 0.001**	0.021*
*SLC4A2* ‐ rs2303937 (G > A)	0.541	0.358	0.818	0.004*	0.037*
*LASP1* ‐ rs525989 (A > C)	0.524	0.336	0.818	0.004*	0.037*
*ARRDC2* ‐ rs7259041 (T > A)	1.911	1.196	3.053	0.007*	0.042*
*CCSER1* ‐ rs6843110 (A > C)	1.690	1.144	2.498	0.008*	0.042*
*PTPN5* ‐ rs1550870 (C > A)	0.598	0.402	0.890	0.011*	0.047*
*RNF222* ‐ rs12601265 (G > A)	1.706	1.108	2.628	0.015*	0.055
*COLEC11* ‐ rs3811527 (A > C)	0.574	0.357	0.921	0.021*	0.059
*ITPR1* ‐ rs2306875 (G > A)	0.589	0.375	0.927	0.022*	0.059
*TMEM11* ‐ rs3744223 (C > A)	0.621	0.411	0.938	0.024*	0.059
*C3* ‐ rs2287845 (G > A)	0.626	0.407	0.963	0.033*	0.069
*COL19A1* ‐ rs7762409 (C > A)	1.578	1.033	2.410	0.035*	0.069
*LINC01118* ‐ rs2289933 (C > A)	1.522	1.028	2.253	0.036*	0.069
*TRIM6* ‐ rs3751006 (A > C)	1.548	1.022	2.345	0.039*	0.070
*SLC23A2* ‐ rs1110277 (A > C)	0.652	0.430	0.986	0.043*	0.071
*ODAD1* ‐ rs8107712 (G > A)	0.669	0.451	0.994	0.046*	0.072
*SNED1* ‐ rs7571117 (T > A)	1.493	0.998	2.233	0.051	0.075
*NPC1* ‐ rs1367083 (C > A)	0.695	0.470	1.028	0.068	0.095
*RAI14* ‐ rs1048944 (G > A)	1.419	0.967	2.083	0.074	0.097
*PODN* ‐ rs1769316 (G > A)	1.416	0.946	2.118	0.091	0.114
*SCN9A* ‐ rs6747673 (A > C)	0.759	0.513	1.124	0.169	0.201
*PLA2G4D* ‐ rs4924618 (A > C)	1.288	0.851	1.949	0.231	0.262
*NRP2* ‐ rs849526 (T > A)	0.794	0.529	1.194	0.268	0.291
*PPARGC1A* ‐ rs3755863 (C > A)	1.251	0.827	1.890	0.289	0.301
*GAD2* ‐ rs2839671 (G > A)	1.240	0.774	1.986	0.372	0.372

*Note:* Caption: Odds ratios (OR). 95% confidence intervals (L95–U95). nominal *p*‐values. and false discovery rate (FDR)‐adjusted *p*‐values are shown for each SNP under an additive genetic model. OR > 1 indicates increased odds of severe disease. whereas OR < 1 indicates decreased odds within the analyzed cohort. Values in bold and marked with an * indicate statistical significance (*p*‐value < 0.05 and/or FDR‐BH < 0.05). Associations should be interpreted in the context of multiple testing and the exploratory design of the study.

### 3.5 In Silico Functional Prediction Using CADD

We investigated the PHRED scale scores using CADD v1.6 software (Supporting Information: Table 5). Most of the variants described here presented PHRED scores below 10. However, some variants presented moderately high scores, among which: *NRP2* ‐ rs849526 (PHRED = 34), *ITPR1* ‐ rs2306875 (PHRED = 20.7), *ARRDC2* ‐ rs7259041 (PHRED = 18.15), *RAI14* ‐ rs1048944 (PHRED = 16.40). All in silico predictions reported here provide contextual support but cannot be interpreted as direct causal evidence.

### 3.6 Regulatory Annotation Using GTEx v8 eQTL Data

To investigate the potential regulatory effects of our prioritized variants, we consulted eQTL and sQTL data from GTEx v8. Eight loci demonstrated significant genotype‐dependent expression differences in one or more tissues of interest for COVID‐19 (Supporting Information: Table 6).

Expression‐associated signals were observed for *PODN ‐* rs1769316 (lung), *GAD2 ‐* rs2839671 (cerebellum), *COL19A1 ‐* rs7762409 (cerebellum), *NPC1 ‐* rs1367083 (lung, cardiovascular and brain regions), *ODAD1 ‐* rs8107712 (whole blood and lung), *NRP2 ‐* rs849526 (lung). GTEx annotations reflect baseline regulatory variation in non‐infected individuals and may not capture infection‐specific regulatory dynamics.

## 4 Discussion

### 4.1 Association of Sociodemographic Factors and Comorbidities with COVID Severity

In the analyzed cohort (*N* = 325), the distribution of clinical severity and its main determinants was consistent with patterns previously described in the literature. The association between male sex and disease severity corroborates findings from large population‐based studies [13]. The association of obesity, diabetes mellitus, and chronic cardiovascular and lung diseases with severity further supports the role of preexisting cardiometabolic and respiratory vulnerability in clinical progression [14].

Additionally, the association between lower educational attainment and disease severity points to the contribution of social determinants of health, aligning with evidence of socioeconomic risk gradients in COVID‐19 outcomes [15]. It is well recognized that clinical and demographic factors can influence the risk gradients observed in COVID‐19; however, they are not able to fully explain the observed interindividual variability.

### 4.2 Biological Characterization of Genes Prioritized by SHAP

Exploratory pathway enrichment analysis of the genes selected by SVM (*n* = 25) revealed processes related to mitochondria, neuroimmune signaling pathways, and complement activation. After adjusting for age, sex, major components of ancestry (PCAs) and statistically significant comorbidities, some variants remained significant even after FDR correction (Table 2). More comprehensive details are presented in Supporting Information: Tables 1–4.

### 4.3 Mitochondrial Metabolism Axis

The selection of genes such as *NPC1* ‐ rs1367083, *TMEM11* ‐ rs3744223, *SLC4A*2 ‐ rs2303937, *PPARGC1A* ‐ rs3755863 and *SLC23A2* ‐ rs1110277 aligns with previous literature implicating immunometabolic pathways, immunometabolic involvement related to lipid homeostasis, mitochondrial structure, intracellular pH dynamics and antioxidant defense mechanisms in COVID‐19 [16, 17, 18, 19, 20].

Among the prioritized genes, *NPC1* has been implicated in infections caused by enveloped viruses, including SARS‐CoV‐2, and may reflect potential involvement in endolysosomal cholesterol trafficking and pH regulation [16]. Experimental inhibition of NPC1 protein has been associated with reduced viral entry, supporting its potential relevance in host–virus interactions [21].

Severe COVID‐19 is also accompanied by mitochondrial dysfunction and immune reprogramming, drawing attention to regulators of mitochondrial architecture and bioenergetic efficiency [22]. In the context of mitochondrial dysfunction, the *TMEM11* gene has been associated with the organization of mitochondrial cristae and the bioenergetics of immune cells. Furthermore, disruption of mitochondrial architecture appears to contribute to redox imbalance under inflammatory stress conditions [17] such as COVID‐19. In severe inflammatory states, mitochondrial structural alterations have been associated with redox imbalance and altered bioenergetics; phenomena also described in severe COVID‐19 [23].

Alterations in pH control can affect key metabolic pathways and redox balance [24]. Under this perspective, the selection of *SLC4A2* is noteworthy, as it regulates cytosolic pH [18]. Sustained pH dysregulation may impair antioxidant systems and increase susceptibility to oxidative stress under persistent inflammatory conditions [25], observed in COVID‐19 [23].

Notably, the variant *SLC4A2 ‐* rs2303937 remained associated with decreased odds of severity after FDR correction (Table 2). The *TMEM11 ‐* rs3744223 variant also demonstrated moderate CADD scores, while the *NPC1 ‐* rs1367083 variant showed widespread eQTL signals across lung and cardiovascular tissues in GTEx. These observations indicate that part of the signal identified in this axis lies within regions of potential regulatory relevance, although experimental validation is required.

In the context of bioenergetic stress and redox imbalance, we also identified *PPARGC1A ‐ rs3755863* in our analyses. Previous studies indicate that PPARGC1A protein interacts with transcription factors such as NRF1, NRF2, and ERRα, regulating the expression of components of the electron transport chain (ETC). Alterations in the ETC are associated with increased oxidative stress and mitochondrial senescence [19].

The control of cellular oxidative stress is partially mediated by antioxidant vitamins. The *SLC23A2* gene, which regulates intracellular vitamin C transport, may influence antioxidant defense mechanisms under inflammatory stress conditions [20].

Even though clinical evidence regarding vitamin C supplementation in COVID‐19 remains inconclusive, reduced circulating levels have been reported in severe cases [26]. These observations raise the possibility that both the inflammatory balance and different responses to oxidative stress may be influenced by variability in vitamin C transport mediated by *SLC23A2* (SVCT2) in conjunction with other factors.

To better visualize how our results might act within a shared biological cascade, we illustrate a possible functional integration in Figure 2. We emphasize the importance of looking at the prioritized genes not independently, but rather through their participation in pathways that may overlap and converge towards distinct outcomes.

**Figure 2:**
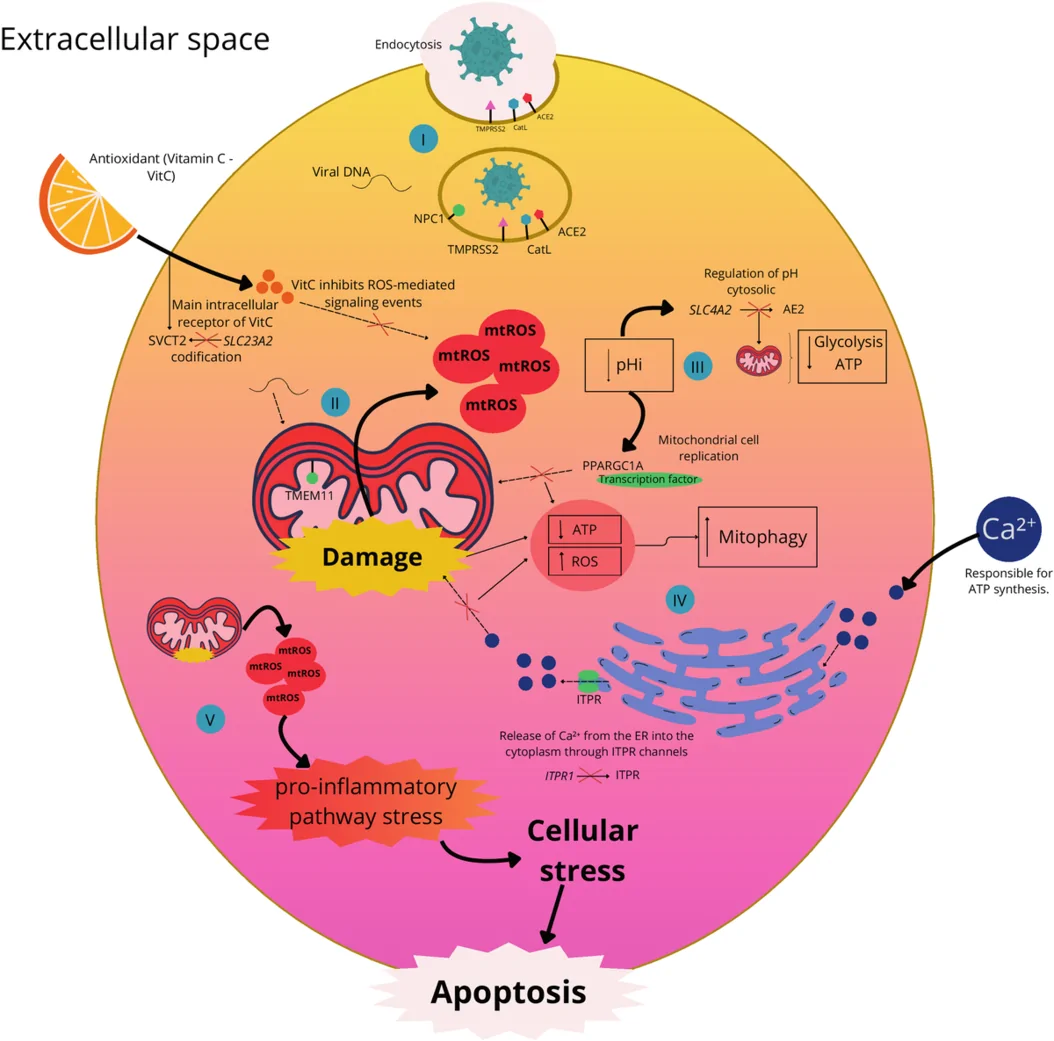
Conceptual framework illustrating biological pathways represented by prioritized genes in severe coronavirus disease 2019 (COVID‐19). The diagram presented here illustrates the cellular pathways that have been previously linked to severe acute respiratory syndrome coronavirus 2 (SARS‐CoV‐2) infection and are represented by genes prioritized in our analyses. We intend only to create a hypothesis‐generating framework and not necessarily a validated mechanistic sequence. A priori, we highlight: (I) Viral entry and endolysosomal processes. SARS‐CoV‐2 enters the cell through receptors such as ACE2/TMPRSS2/CatL, and this entry pathway has already been associated with alterations in endolysosomal trafficking and cholesterol metabolism. The genes involved in this pathway may reflect host factors that influence intracellular lipid and pH regulation, and therefore we highlight the *NPC1* gene here. (II) Mitochondrial function and oxidative stress. Previous research has already observed associations between mitochondrial structural alterations and redox imbalance. We highlight genes that mainly participate in mitochondrial architecture, biogenesis, and antioxidant transport (T*MEM11, PPARGC1A, and SLC23A2*). All these pathways can influence cellular responses to inflammatory stress. (III) Bioenergetics and regulation of intracellular pH. Still within the mitochondrial context, it is known that cellular energy production depends on coordinated mitochondrial activity in conjunction with cytosolic pH homeostasis. Our hypothesis is that genes involved in pH regulation may interact with metabolic pathways that can influence the oxidative state in inflammatory conditions. (IV) Calcium signaling and metabolic integration. Calcium flow between the endoplasmic reticulum and mitochondria has already been implicated in metabolic regulation and stress signaling. This flow is mediated, in part, by the ITPR1 receptor, and alterations in calcium dynamics have been described in inflammatory states, interconnected with mitochondrial function. (V) Inflammatory activation and cellular stress responses. Reactive oxygen species, mainly from mitochondrial processes of metabolic imbalance and ionic signaling, have already been associated with the activation of inflammatory pathways in severe infections. In general, we understand that the genes represented may operate within this broader network of host response. For a better understanding of the figure, we use solid lines to indicate biological relationships described in the literature, and dashed lines to represent possible interactions resulting from the overlap of pathways.

### 4.4 Neuroinflammation and Neuroimmune Signaling Axis

From the perspective of immunometabolic dysregulation, autophagy represents an important host defense mechanism. Represented in Figure 2, ITPR1 protein regulates Ca^2+^ transfer to mitochondria, and alterations in mitochondrial Ca^2+^ flux can influence, redox balance, and autophagy‐dependent pathways [27, 28].

Consistent with this, *ITPR1* ‐ rs2306875, showed decreased odds of severe disease in regression analysis, suggesting a possible modulatory role of calcium signaling in clinical variability within this cohort. Furthermore, the variant displayed moderately elevated CADD scores (Supporting Information: S5).

The Ca^2+^ signaling can activate cascades such as Raf/MEK/ERK [29]. Activation of the Raf/MEK/ERK pathway in the CNS is controlled by phosphatase‐dependent processes, primarily through the non‐receptor tyrosine phosphatase PTPN5 (STEP). Recent studies have shown that reduced PTPN5 protein expression or activity appears to be associated with a sustained increase in Ca^2+^‐dependent signaling and neuronal remodeling [30].

Sustained ionic dysregulation may affect excitatory–inhibitory balance in neural circuits. *GAD2*, which encodes glutamate decarboxylase involved in GABA synthesis, is functionally linked to intracellular Ca^2+^ dynamics [31]. In our analysis, rs2839671 was prioritized by the ML framework and showed genotype‐associated expression differences in cerebellar tissue in GTEx, suggesting potential regulatory variation in neural contexts.

While transient elevations in Ca^2+^ appear to activate compensatory mechanisms for GABA release, persistent elevations, associated with inflammation and prolonged activation of the ERK pathway, compromise GABAergic signaling and favor imbalances in sensory and circadian circuits [32]. Such imbalance has been associated with symptoms such as anxiety, insomnia, psychomotor agitation, and sensory hypersensitivity, frequently reported during the acute phase of infection [33].

Ionic imbalance may influence sodium channel activity. Consistent with this, functional studies have demonstrated that the proper activity of the voltage‐gated sodium channel NaV1.7, encoded by the *SCN9A* gene, is fundamental for the propagation of action potentials in the olfactory system. Thus, its dysregulation is sufficient to interrupt electrical conduction along the olfactory nerve and result in dysfunctions such as anosmia [34].

The observations described in this section are consistent with the possible involvement of genes related to ion channels in the variability of neuroimmune signaling during severe infections. However, no direct functional inference can be made about a functional link between systemic inflammation and neurosensory symptoms, making it purely speculative.

### 4.5 Immune and Inflammatory Axis

The bioenergetic dysregulation discussed earlier may represent a relevant metabolic context for modulating the immune response. In this sense, the immune and inflammatory axis presented in this topic can be considered a possible functional interface between metabolic alterations and inflammatory processes observed in COVID‐19.

The prioritization of genes involved in post‐translational regulation of innate immunity may be compatible with a potential role of E3 ubiquitin ligases in modulating antiviral responses. The *TRIM6* gene, a regulator of type I interferon (IFN‐I) signaling, has been experimentally implicated in SARS‐CoV‐2 replication through lysine‐29 (K29)‐linked ubiquitination of the viral nucleocapsid [35].

In the present cohort, the intronic rs3751006 variant in *TRIM6* showed a negative directional contribution in SHAP analysis, a finding that does not fully align with previously described pro‐viral mechanisms. However, this observation should be interpreted cautiously. It is possible that rs3751006 may be linked to regulatory or context‐dependent effects on *TRIM6* expression or splicing, although no functional evidence currently supports this hypothesis.

Similarly, another ubiquitin ligase identified in our analysis was *RNF222*. The rs1260125 variant remained associated with disease severity after regression analysis. RNF222 belongs to the family of E3 ubiquitin ligases with a RING finger domain, involved in innate immunity and interferon signaling. Similar ligases, such as RNF220, regulate IFN‐STAT1 pathways through ubiquitination [36], although the specific role of RNF222 remains uncertain.

Furthermore, the *ARRDC2* gene emerged as an additional candidate, as members of the α‐arrestin family function as adaptors that recruit ubiquitin E3 ligases [37]. Although not directly linked to SARS‐CoV‐2, processes involving E3 ligases may have an indirect effect on the modulation of the complement system [37].

The intensification of the inflammatory response in COVID‐19 also appears to be related to reactions that generate the C3 protease. Some previous experimental models have described that the absence of C3 significantly reduced lung injury, neutrophil recruitment, and cytokine production in severely ill patients [38]. In our cohort, the decreased odds of severe disease observed for *C3* ‐ rs2287845, observed in the regression analysis may suggest variability in complement‐related pathways; however, the direction and functional relevance of this association remain uncertain.

Similarly, *COLEC11*, a gene responsible for encoding lectin pathway recognition molecules, has been linked to the severity of COVID‐19, although viral resistance to complement‐mediated synthesis suggests a complex dynamic of immune evasion [39].

Beyond the complement system, the severity of COVID‐19 involves subsequent inflammatory pathways that determine the extent of tissue damage. Activation of *PLA2G4D* appears to favor the production of pro‐inflammatory eicosanoids associated with increased vascular permeability, and pulmonary thrombosis [40]. These mediators act synergistically with complement, amplifying leukocyte recruitment and sustained inflammation. Mechanisms already observed in severe COVID‐19 [41].

As this inflammatory response self‐amplifies, processes involved in its containment begin to influence the persistence of tissue damage. This is how neuropilin‐2 (*NRP2*) can negatively modulate inflammatory signaling through Toll‐like receptors [42]. Loss of NRP2 protein function results in persistent neutrophilia and failure of efferocytosis. Alterations in *NRP2* could hypothetically be linked to inflammatory regulation pathways described in severe COVID‐19, although direct mechanistic involvement cannot be determined from the present data [43].

In this immune and inflammatory axis, *ARRDC2* ‐ rs7259041 remained associated with increased odds of severe disease after FDR correction and demonstrated moderately elevated CADD scores, indicating potential functional constraint. The intron variant *TRIM6* ‐ rs3751006 and *NRP2* ‐ rs849526 showed genotype‐dependent expression differences in GTEx across lung, and immune‐related tissues. Although these findings do not confirm functional modulation by SARS‐CoV‐2, several loci prioritized by the classifier appear to be embedded in regulatory networks relevant to innate immunity.

### 4.6 Epithelial Integrity and Structural Remodeling Axis

In the respiratory epithelium, transmembrane mucins form a glycosylated barrier that limits the adhesion and entry of SARS‐CoV‐2 [44]. Elevated *MUC16* expression has been reported in severe cases of COVID‐19, suggesting that excessive mucosal responses may contribute to impaired ventilation and barrier dysfunction [45].

In this context, genes such as *PODN* and *SNED1*, involved in cell‐matrix interaction and tissue remodeling, could be compatible with pro‐fibrogenic responses, consistent with the diffuse alveolar damage and pulmonary fibrosis observed in severe and post‐acute COVID‐19 [45, 46].

Airway defense also depends on ciliary integrity, and the selection of *ODAD1*, a gene involved in ciliary stability, aligns with reports of ciliary dysfunction during SARS‐CoV‐2 infection [47].

Similarly, genes involved in cytoskeletal organization and junctional stability (*RAI14, CCSER1*) reinforce the concept of epithelial structural fragility, while the protective signal observed for *LASP1* ‐ rs525989 suggests that the preservation of actin organization may attenuate barrier disruption [48].

The inclusion of *COL19A1* and the long non‐coding RNA *LINC01118* expands this structural axis, indicating that extracellular, microvascular, and epigenetic mechanisms contribute in a coordinated manner to the dysregulation of tissue repair and the clinical heterogeneity observed in COVID‐19 [49].

Consistent with these functional contexts, *MUC16* ‐ rs12462963, and *CCSER1* ‐ rs6843110 were associated with disease severity after FDR correction. In addition, variants in *COL19A1 ‐* rs7762409, *PODN* ‐ rs1769316, and *ODAD1 ‐* rs8107712 showed genotype‐dependent expression differences in GTEx, providing regulatory context for the structural pathways discussed here. These structural alterations likely interact with broader inflammatory and metabolic dysregulation observed in severe COVID‐19 [50].

### 4.7 Translacional Implications

The results obtained here suggest that, instead of isolated variants, genetic panels based on metabolic pathways can guide future investigations driven by hypotheses about how the host responds in terms of metabolic pathways. Our findings are exploratory in nature, but point to possible biological pathways that deserve further investigation through mechanistic studies. It is not possible to infer any clinical application of these findings without validation in larger, well‐defined cohorts.

### 4.8 Limitations

When interpreting the results, attention should be paid to the limitations of this study. Feature selection was performed using a supervised approach across the entire cohort. Although a train‐test split was applied for performance estimation, the primary objective was exploratory variant prioritization, not the validation of predictive models. Therefore, related performance metrics should be interpreted with caution. Nevertheless, this feature is also advantageous, as it contributes something new to the literature by relating a distinct and underrepresented group in global genomic studies. These aspects presented here, instead of diminishing the importance of our results, highlight the need to validate the results in different landscapes and through longitudinal studies, which may serve as a basis both for understanding SARS‐CoV‐2 infection and for addressing future pandemics. Another factor to consider is the temporal scope of the cohort (2020–2024), which spans multiple SARS‐CoV‐2 variant waves with differing transmissibility and virulence (Supporting Information: Figure 1). Therefore, we cannot exclude the possibility that changes in circulating variants influenced disease severity.

## 5 Conclusion

In summary, this study provides a broad and detailed picture of how host genomic variability affects the severity of COVID‐19 in an admixed Brazilian cohort. We found a group of variants distributed across connected biological axes, such as mitochondrial bioenergetics, immune regulation, neuroimmune signaling, and epithelial structural integrity. These findings were derived through machine‐learning–based prioritization, regression modeling with ancestry adjustment, and in silico functional annotation. Our results suggest pathway‐level interactions involving immunometabolic imbalance, complement activation, neuronal excitability, and tissue remodeling may all be linked at the pathway level. It is essential to recognize these connections within a framework for formulating hypotheses, as they do not establish causal mechanisms. Combining statistical association with functional prediction (CADD) and baseline regulatory evidence (GTEx) makes biological sense, but it is important to note that these datasets cannot be used to make conclusions about infection‐specific molecular dynamics. The current results highlight the complexity of the host genetic architecture underlying severe COVID‐19. They also stress the need for replication in separate, ethnically diverse groups and functional validation studies to make clear how the prioritized loci work.

## Supporting information


Supporting File 1

Supporting File 2

Supporting File 3

Supporting File 4

## Data Availability

The data that support the findings of this study are available from the corresponding author upon reasonable request.
