# Supplementary material for: Deciphering Host Genomic Pathway Landscapes in Severe COVID‐19 by Exome Sequencing of a Large Admixed Brazilian Cohort
Source: J Med Virol. 2026 Nov 2;98(11):e71193. doi: 10.1002/jmv.71193 (PMC13726577; doi:10.1002/jmv.71193)
Supplement: Supplementary file 1 [file JMV-98-e71193-s004.docx]

**Supplementary Figure 1. Monthly distribution of COVID-19 infections by clinical severity and predominant SARS-CoV-2 variant periods.**

**Supplementary Figure 1.** Monthly counts of COVID-19 infections stratified by clinical spectrum (Mild, blue; Severe, red) across the study period (January 2020–February 2024). Vertical shaded regions indicate predominant SARS-CoV-2 variant periods documented in Brazil: **Wave 1** (original lineages B.1.1.28/B.1.1.33; Mar–Dec 2020); **Wave 2** (P.1/Gamma dominant, with co-circulation of P.2 and Alpha; Jan–Jul 2021); **Wave 3** (Delta B.1.617.2+AY.*, with emergence of Omicron BA.1.*; Aug–Dec 2021); **Wave 4** (Omicron BA.2.* dominant; Jan 2022–onward). The peak of severe cases in March–April 2021 coincides with the Gamma (P.1) variant surge. Mild cases predominate across Omicron-dominant periods (2022–2024). Dashes (—) indicate months with zero cases in the cohort. Data derived from column AS (Date of Infection) and column F (Clinical Spectrum) of the unified cohort dataset (n = 325).
